# Impact of In-Home Repair Services on Physical and Mental Harm among Essential Workers: The Mediating Effect of Fear and Moderating Effect of Types of Employment

**DOI:** 10.3390/bs12120497

**Published:** 2022-12-06

**Authors:** Jee-Hyun Hwang, Hye-Sun Jung

**Affiliations:** 1 School of Nursing, University of California San Francisco, San Francisco, CA 94143, USA; 2 College of Medicine, Catholic University of Korea, Seoul 06591, Republic of Korea

**Keywords:** essential workers, in-home repair services, fear, severity of physical and mental harm, types of employment

## Abstract

This study attempted to establish a hypothetical model describing the severity of physical and mental harm among essential workers during the COVID-19 pandemic. To this end, the mediating effect of fear on the relationship between in-home services and the severity of physical and mental harm was analyzed. Moreover, this study utilized multigroup path analysis to examine differences according to the type of employment. Thus, data from all 502 participants were included in the final analysis. The study found that in-home service did not have a direct effect on fear in the path model for the permanent employment group, but did have a direct effect on fear in the path model for the non-permanent employment group. The implications for the field are that the following is required: an anti-infection system should be incorporated at workplaces; employment stability should be provided for essential workers; providing systematic support, such as professional counseling to alleviate negative emotional responses, should be considered.

## 1. Introduction

In December 2019, patients with severe pneumonia of an unknown cause were re-ported in Wuhan, Hubei, China [1]. The first case of coronavirus disease 2019 (COVID-19) in Korea was confirmed on 20 January 2020 [2]. The World Health Organization declared the disease a pandemic on 11 March 2020 [1]. Countries around the world have implemented containment measures, such as imposing restrictions on travel and the use of certain facilities, to prevent the spread of COVID-19 [3]. Amid the prolonged pandemic, essential workers have faced a markedly increased workload, partly attributable to the added work related to anti-infection measures [4].

The COVID-19 pandemic has shed light on our heavy dependence on essential workers [5]. Despite knowing that essential workers are indispensable, they are often unprotected and are the most vulnerable [6]. Hence, it is important to ensure the safety of essential workers and to adopt systems and provide support so as to protect their health, even after the crisis is resolved [5].

In the advent of the pandemic, the importance of healthcare and other services essential to maintaining our daily lives and economic activities have been highlighted worldwide. In particular, tasks necessary for maintaining daily life such as delivery services, repair services, and street cleaning were highlighted [7]. Thus, it is necessary to pay attention to essential workers who continue to provide in-person services during the COVID-19 crisis [8].

In particular, essential workers such as appliance installation and repair technicians are required to visit customers’ homes or offices and are thus at an elevated risk of COVID-19 infection. Extended durations of face-to-face contact with customers during in-home or in-office services increase the technicians’ fears [9]. The fear of COVID-19 encompasses fear of the infection itself, as well as the fear of social stigma and anxiety about potential changes in the quality of life [10]. Unsurprisingly, the fear of infection due to face-to-face contact has increased significantly since the spread of the COVID-19 infection [11].

The fear of COVID-19 affects individuals’ mental health, which in turn poses long-term problems in the labor force [12]. Moreover, the fear of COVID-19 influences perceived physical and mental health [13,14,15]. It is the most common emotion prevalent among individuals during the COVID-19 outbreak and is also a predictor of physical and mental health status [16].

Exposure to various work-related hazards varies depending on the type of employment. Compared to permanent workers, fixed-term or contract workers are more likely to be exposed to hazards [17]. The type of employment significantly predicts an individual’s self-rated health, with employment stability a key requirement for a healthy life [18]. The working conditions of essential workers are incommensurate with the value of their labor during the pandemic [19]. Thus, society must protect essential workers and resolve unfair labor conditions [19].

In this context, this study investigated the impact of in-home services on the severity of physical and mental harm through the mediation of fear, according to the type of employment, in essential workers. To this end, the mediating effect of fear on the relationship between in-home services and the severity of physical and mental harm was analyzed. Moreover, this study utilized multigroup path analysis to examine differences according to the type of employment.

The findings of this study will be useful as foundational data for the understanding of physical and mental harm among essential workers, as well as the development of policies and systems for preventing such harm. Furthermore, the results will have valuable implications in that they shed light on the differences of effects according to the type of employment and therefore can help adjust the direction of policies targeting the prevention of physical and mental harm among these workers.

## 2. Materials and Methods

### 2.1. Hypothesis Development

**Figure 1:**
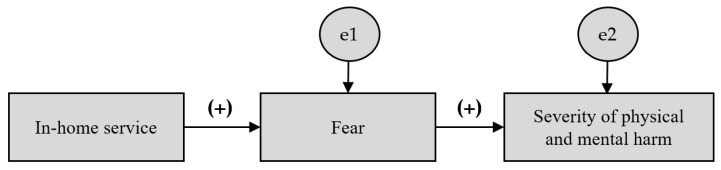
Conceptual framework for this study. Analysis of the difference between the group as a whole and the group with permanent employment and the group with non-permanent employment.

This study attempted to establish a hypothetical model describing the severity of physical and mental harm among essential workers during the COVID-19 pandemic. With reference to previous findings that in-home services contribute to physical and mental harm [9,10], this study tested the hypothesis using multigroup path analysis. The mediating effect of fear on the relationship between in-home services and physical and mental harm in permanently employed and contract-employed workers was analyzed using multigroup path analysis. A hypothetical path model was established with the type of employment as a moderator, with reference to previous findings that employment instability has an adverse impact on self-rated health [20,21,22] (Figure 1).

Our hypotheses could be:

**Hypothesis** **1:** *More in-home services mediate fear, increasing the severity of physical and mental harm.*

**Hypothesis** **2:** *Differences in pathways exist between types of employment groups.*

**Hypothesis** **3:** *The permanent employment group has a small effect of in-home services on the severity of physical and mental harm.*

### 2.2. Study Sample

Appliance installation and repair service technicians in Korea were enrolled in this study. The subjects of this study had the same job type. Essential workers who provided informed consent to participate in the study were convenience sampled. Data were collected via an online survey using structured questionnaires. There were no missing responses, as the questionnaire could not be completed without providing a response to all items. Thus, data from all 502 participants were included in the final analysis.

### 2.3. Data and Empirical Method

The data collection of this study was conducted from 24 May to 31 May 2021. The researcher identified the current status of appliance installation and repair service technicians through interviews with the labor union. The researcher explained the purpose and method of this study to the labor union and workplace. The researcher received prior approval for the research process through a direct visit to the company or by a phone call. The purpose and method of the study, procedure, anonymity guarantee, and possibility of withdrawal during the study were explained to the study subjects in writing, ensuring that there were no disadvantages. Subjects who understood this and voluntarily agreed to it conducted an online survey. The survey consisted of 7 general and occupational characteristics of the study participants, 9 questions about in-home repair services and COVID-19, 8 questions about the risk of COVID-19, and 12 questions about workplace quarantine measures for infectious diseases.

### 2.4. Measurements and Variable Definitions

#### 2.4.1. In-Home Service

An in-home service was defined as the average number of places visited per day to provide appliance installation and repair services. The response was a continuous variable, with a higher value indicating more places visited.

#### 2.4.2. Fear

Fear was assessed using the fear subscale in a previous study about COVID-19 risk awareness [23], with eight items pertaining to COVID-19: fear about potential confirmed cases at work; fear of contracting the infection; fear of going into quarantine; fear of contracting the infection and being asymptomatic; fear of potential disadvantages for using sick days at work; fear of criticism for contracting the infection; fear of having people who do not report their symptoms; and fear of meeting an asymptomatic patient through work. Each item was rated on a five-point Likert scale from 1 (“strongly agree”) to 5 (“strongly disagree”), with the responses reverse-coded. A higher score indicates a greater fear of COVID-19. The reliability (Cronbach’s ⍺) of the tool was 0.909.

#### 2.4.3. Severity of Physical and Mental Harm

The severity of physical and mental harm was assessed using a numeric rating scale (NRS) from 0 (“no harm at all”) to 10 (“very severe harm”). A higher score indicates more severe physical and mental harm.

#### 2.4.4. Type of Employment

The type of employment was chosen from “permanent employment”, “contract employment”, and “other”. Those who chose “permanent employment” were assigned to the permanent employment group, and those who chose “contract employment” or “other” were assigned to the non-permanent employment group.

### 2.5. Statistical Analysis

The collected data were analyzed using the SPSS (IBM SPSS Statistics for Windows, Version 23.0. Armonk, NY, USA: IBM Corp.) and SPSS Amos 20.0 software. The reliability of the instruments was evaluated using Cronbach’s α coefficient. Participants’ general characteristics, in-home service, fear, physical and mental harm, and type of employment were analyzed with descriptive statistics. The correlations among the major study variables were analyzed with Pearson correlation analysis. The fit of the developed model was tested using SPSS Amos 20.0 software, with the Tucker–Lewis Index (TLI), Comparative Fit Index (CFI), and Root Mean Square Error of Approximation (RMSEA). To examine the paths within the model, the statistical significance of β and *p* values were examined for each path coefficient. Indirect effects were tested using bootstrapping. The differences in the associations among the variables, according to the type of employment, were analyzed with multigroup analyses, based on Δχ^2^ statistic and *p* values. The significance of each path in the permanent employment group and non-permanent employment group was examined using β and *p* values.

### 2.6. Ethical Considerations

This study was approved by the Institutional Review Board of the Catholic University of Korea (IRB; MC20QISI0123) and performed in accordance with the Declaration of Helsinki.

## 3. Results

### 3.1. General Characteristics of the Subjects

The general characteristics of the subjects are presented in Table 1. The majority of participants were male (97.4%). The mean age was 42.9 ± 0.29 years. The majority of workers were employed in businesses with <50 employees (73.9%). The mean length of employment was 12.9 ± 0.56 years. The mean weekly working time was 41.4 ± 0.78 h, with the most common weekly working time being ≤40 h (40.6%), followed by ≥52 h (32.1%) and 41–51 h (27.3%) (Table 1).

### 3.2. Descriptive Statistics of Measurement Variables

The levels of the major study variables were presented with descriptive statistics. The mean number of places visited for services per day was 12.85 (0–30). The mean fear score was 35.38 (8–40). The mean physical and mental harm score was 9.01 (0–10). Regarding the type of employment, 404 had a permanent job (80.5%), while 98 (19.5%) had a non-permanent job.

### 3.3. Correlations among Measurement Variables

The correlations among the major study variables were analyzed with Pearson correlation analysis. In-home service was significantly positively correlated with fear (r = 0.110, *p* = 0.014) and with the severity of physical and mental harm (r = 0.115, *p* = 0.010). Fear was significantly positively correlated with the severity of physical and mental harm (r = 0.505, *p* = 0.000) (Table 2).

### 3.4. Path Model Fit

This study aimed to investigate the effects of in-home services on the severity of physical and mental harm through the mediation of fear. The following path model was designed for this purpose.

The fit of the developed model was tested using TLI, CFI, and RMSEA, which are fit indices used for large sample sizes [24]. In general, a TLI and CFI of 0.90 or higher and a RMSEA of below 0.08 are considered to indicate a good fit [25]. The path model of this study was found to have a good fit with a TLI = 0.972, CFI = 0.991, and RMSEA = 0.053.

### 3.5. Significance of Path Coefficients

The associations among the variables were analyzed by examining the statistical significance of each path coefficient.

The path from in-home service to fear was significantly positive (β = 0.110, *p* = 0.014), while the path from fear to the severity of physical and mental harm was also significantly positive (β = 0.505, *p* < 0.001). In other words, fear was greater with more places visited to provide in-home services, while the severity of physical and mental harm was greater with greater fear.

### 3.6. Evaluation of Indirect Effects

The indirect effects were tested with bootstrapping. The bootstrapping sample size was set to 500 and significance was tested at a 95% confidence level (CI). The 95% CI for two indirect paths did not contain 0, confirming that the indirect effects are statistically significant.

In-home service had a significant indirect positive effect on the severity of physical and mental harm through a sequential mediation by fear (B = 0.032, *p* < 0.05) (Table 3).

### 3.7. Differences in the Associations among Variables according to the Type of Employment (Multigroup Analysis)

The participants were divided into the permanent employment group and the non-permanent employment group depending on the type of their employment. To analyze the differences in the associations among the variables according to the type of employment, measurement invariance was tested through multigroup confirmatory factor analysis. The fit of the unconstrained model had χ^2^ = 7.331 (*p* = 0.026), TLI = 0.900, CTI = 0.967, and RMSEA = 0.073, confirming measurement invariance across groups. The χ^2^ for the unconstrained, structural weights, and structural intercepts models were not significant (Table 4). In other words, the two groups had an equal model as well as measurement invariance of factor coefficients between the latent and measurement variables, based on which the data were considered suitable for multigroup path analysis (Table 4).

In the permanent employment group, in-home service did not significantly influence fear. On the other hand, in-home service significantly influenced fear in the non-permanent employment group (β = 0.262, *p* = 0.007). The differences in the paths involving in-home service and fear between the two employment type groups were not statistically significant.

**Figure 2:**
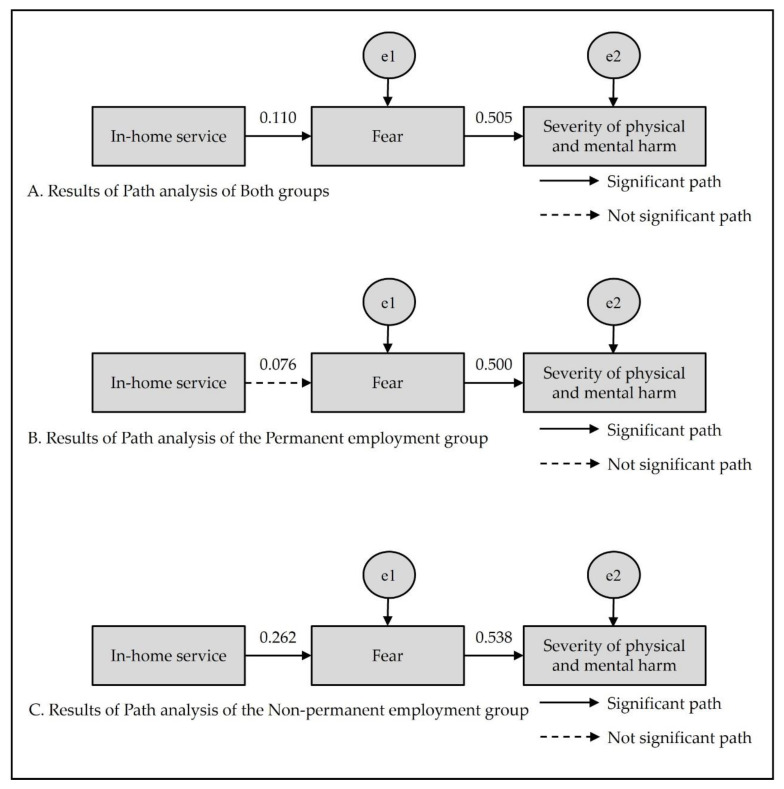
Results of path analysis.

Fear significantly positively influenced the severity of physical and mental harm in both the permanent employment group (β = 0.500, *p* < 0.001) and the non-permanent employment group (β = 0.538, *p* < 0.001). In other words, the severity of physical and mental harm increased with increasing fear in both groups. The differences in the paths involving fear and the severity of physical and mental harm between the two employment type groups were not statistically significant (Table 5) (Figure 2).

## 4. Discussion

This study identified the predictors of the severity of physical and mental harm among essential workers during the COVID-19 pandemic. Moreover, we analyzed the effects of in-home services on the severity of physical and mental harm according to the type of employment and examined the mediating effect of fear on this relationship using multigroup path analysis.

The study population was predominantly male (97.4%) and aged between 40–49 years (51.6%), with a mean age of 42.9 years. In a study on delivery workers [26], the study population was also predominantly male (92.3%), with the greatest number of participants in the 40–49 years age group (31.5%). Our study participants therefore have similar general characteristics to those of delivery workers. The mean weekly working time was 41.36 h in this study, similar to the 43.59 h found in a study on special employment workers [27]. Special employment workers include insurance agents, home-study teachers, quick service delivery drivers, golf caddies, door-to-door salespeople, and designated drivers [27]. The mean length of employment of our study population was 12.9 years, which is longer than the 6.32 years reported among special employment workers [27] and the 5.5 years reported among delivery workers [26]. The longer length of employment may be attributable to the fact that appliance installation and repair services require special and professional skills. The majority of the participants worked in small businesses with fewer than 50 employees (73.9%), while 20.3% worked in large businesses with 300 or more employees. In future, practical policies and systems pertinent to health management and human resources management for appliance installation and repair technicians should be tailored to the size of businesses.

Essential workers refer to those who provide essential services to protect the lives and health of the public and maintain social functions even during a disaster [28]. This means that essential workers must continue providing face-to-face services even during the COVID-19 pandemic [8]. In particular, appliance installation and repair technicians are required to visit customers’ homes or offices in person and are thus at a higher risk for COVID-19 infection.

The results of this study showed that fear among appliance installation and repair technicians increases with a greater number of places visited to provide in-person services. A previous study reported that prolonged face-to-face contact with a customer during in-person services influences workers’ emotional status, such as fear [9]. Thus, anti-infection measures need to be implemented in workplaces for essential workers providing in-person services to prevent fear and contribute to stable and continuous work performance.

In terms of employment type, providing in-home services did not influence fear among permanent employees in contrast to non-permanent employees. This suggests that providing in-home services does not affect the severity of physical and mental harm among permanent employees whose employment is stable. According to a study using the Korean Labor and Income Panel Study (KLIPS) data, the type of employment had a significant effect on self-rated health [18]. Further, the said study explained that employment stability is a key requirement for a healthy life [18]. Several other studies consistently reported that employment stability positively affects self-rated health, including emotional status [20,21,22,29,30]. In this sense, promoting employment stability would be an effective strategy to reduce fear among essential workers and alleviate their physical and mental harm. Thus, given the ongoing pandemic, and in preparation for future pandemics, policies that promote employment stability should be implemented to prevent physical and mental harm and thus promote continuity of work among essential workers.

Physical and mental harm increased with increasing COVID-19-related fear. Regarding the type of employment, fear influenced the severity of physical and mental harm in both employment types. The global spread of COVID-19 provoked a drastic increase of fear among socially isolated and vulnerable populations [12]. Fear has an adverse impact on mental health status, such as causing depression, and can lead to long-term problems in the future labor force [12]. Ahorsu et al. [13] reported that the fear of COVID-19 affects perceived physical and mental health status. Shin [14] also reported that the fear of COVID-19 exacerbates mental health problems. In addition, a US study observed a significant association between the fear of COVID-19 and mental health status among socially vulnerable populations [15]. Hence, COVID-19-related fear can be said to aggravate physical and mental harm. Thus, intervention programs that alleviate emotional responses should be developed and implemented before the fear of a pandemic causes physical and mental damage.

Taken together, in-home services provided by essential workers provoke fear among workers and influence the severity of physical and mental harm through the mediation of such fear. However, we observed that in-home service does not influence fear in the path model for the permanent employment group. This suggests that providing in-home services does not contribute to the severity of physical and mental harm among essential workers if their employment is stable. These results will serve as evidence supporting the benefits of employment stability in essential workers.

## 5. Limitations

The limitation of this study is that the participants were convenience sampled to produce prompt results amid the COVID-19 pandemic. Thus, the findings cannot be generalized to the entire essential worker population. In addition, in this study, the psychosocial aspects of workers were not considered. In future studies, it is hoped that follow-up studies will be conducted considering various environments including psychosocial aspects.

## 6. Conclusions

The path model for the entire study population showed that in-home services influenced the severity of physical and mental harm through the mediation of fear. In-home services did not have a direct effect on fear in the path model for the permanent employment group, but did have a direct effect on fear in the path model for the non-permanent employment group.

Based on the results, the present study suggests the following recommendations. First, an anti-infection system should be incorporated at workplaces for essential workers who provide in-person services to prevent any fear related to the infectious disease. Second, in preparation for potential pandemics in the future, policies should be implemented that ensure employment stability for essential workers to prevent their physical and mental harm and to help maintain continuity of work. Third, to prevent physical and mental harm caused by fear of the pandemic, providing systematic support, such as professional counseling to alleviate negative emotional responses, should be considered.

## Figures and Tables

**Table 1:** General characteristics of the subjects (*n* = 502).

Variables	Category	N (%)	Mean ± SD
Age (years)	<40	152 (30.3)	42.9 ± 0.29
	40–49	259 (51.6)	
	≥50	91 (18.1)	
Sex	Male	489 (97.4)	
	Female	13 (2.6)	
Business size (persons)	Small business (1–49)	371 (73.9)	422 ± 82.79
	Medium business (50–299)	29 (5.8)	
	Large business (300 or more)	102 (20.3)	
Length of employment (years)	1–4	138 (27.5)	12.9 ± 0.56
	5–9	129 (25.7)	
	10–19	149 (29.7)	
	≥20	86 (17.1)	
Working time (hours per week)	≤40	204 (40.6)	41.4 ± 0.78
	41–51	137 (27.3)	
	≥52	161 (32.1)	

**Table 2:** Results of path analysis.

Path			B	SE	β	C.R.	*p*
In-home service	→	Fear	0.169	0.068	0.110	2.467	0.014
Fear	→	Severity of physical and mental harm	0.190	0.015	0.505	13.084	<0.001

C.R.—Critical Ratio; SE—Standard Error.

**Table 3:** Indirect effect of independent variable on the severity of physical and mental harm.

Path	Indirect Effect	SE	95% CI		*p*
			LLCI	ULCI	
In-home service → Fear → Severity of physical and mental harm	0.032	0.017	0.003	0.068	<0.050

SE—Standard Error; LLCI—Low Limit Confidence Interval; ULCI—Upper Limit Confidence Interval.

**Table 4:** Multigroup analysis according to type of employment.

Model	χ^2^	df	TLI	CFI	RMSEA	Δχ^2^	Δdf	*p*
Unconstrained	7.331	2	0.900	0.967	0.073			
Structural weights	10.255	4	0.941	0.961	0.056	2.924	2	0.232
Structural intercepts	13.033	6	0.956	0.956	0.048	5.702	4	0.223

CFI—Comparative Fit Index; TLI—Tucker-Lewis Index; RMSEA—Root Mean Square Error of Approximation.

**Table 5:** Results of path analysis according to type of employment.

Path			Type of Employment						Critical Ratios
			Permanent Employment			Non-Permanent Employment			
			B	β.	*p*	B	β.	*p*	
In-home service	→	Fear	0.117	0.076	0.124	0.410	0.262	0.007	1.714
Fear	→	Severity of physical and mental harm	0.191	0.500	<0.001	0.187	0.538	<0.001	−0.123

## Data Availability

Not applicable.
